# COVID-19 pandemic: challenges and opportunities for the Greek health care system

**DOI:** 10.1017/ipm.2020.35

**Published:** 2020-05-14

**Authors:** Ioanna Giannopoulou, George O. Tsobanoglou

**Affiliations:** 1 National and Kapodistrian University of Athens, 2nd Department of Psychiatry Attikon University Hospital, Athens, Greece; 2 University of Aegean, Department of Sociology, Mytilene, Greece

**Keywords:** COVID-19, primary health care, health systems, Greece, impact

## Abstract

After coming out of the state debt crisis, Greece is facing yet another crisis – that of the COVID-19 pandemic. The key challenges facing the organizational structure and function of the Greek public health system in order to meet the populations’ health needs are discussed. Social distancing, through imposed national lockdown very early in the pandemic, has been a key emergency public health measure that has saved lives. However, the system needs to enhance its capacity, through strengthening primary health and social support care, to be able to meet existing unmet health needs, the impact of the pandemic on mental health, as well as to tackle future new waves of outbreak. The related changes in health service provisions in response to the COVID-19 pandemic call for developing new models and novel approaches for delivering effective mental health services.

## Background

Public health expenditure in Greece stands at 5% of GDP, compared to an EU average of 7.2%. Formal or informal out-of-pocket payments comprises 35% of total health spending, more than double the EU average (15%). The largest share of private health expenditure (over 90%) for privately purchased services rather than co-payments (OECD, 2017). The Greek health system is defined by a relatively weak primary health care (PHC) in terms of access, care integration, and continuity in care (Kringos *et al.* 2013). One-to two-thirds of patients attend hospital Emergency Departments with problems that could be managed at PHC level (Tountas *et al.* 2020). Despite several attempts, since the early 2000s, to strengthen and standardize the PHC, Greece is still struggling to attain a sustainable policy-based model of integrated services (Sifaki-Pistolla *et al.* 2017).

Since 2010, the public health care system has been severely affected by the austerity measures driven by Troika. High levels of unmet health needs were reported among the unemployed (21.5%) and those in the low-income groups (34.3% in lowest income *v*. 0.4% in the highest income quintile) (Baeten *et al.* 2018). Against this background, the government refocused its efforts on improving national health care services by enacting two pieces of legislation in 2014 and 2017, with a focus on restructuring PHC and establishing family physician-led care pathways. In addition, in 2016, the health coverage was extended to the uninsured (approximately 2.2 million), who however continued to access mainly hospital-based health services (OECD, 2017).

Citizen’s participation in the PHC system was driven by ‘obligatory’ registration with a family physician (general practitioner, internist, pediatrician) operating at any of the PHC facilities or with contracted private practitioners. However, due to the inadequate financing of the system, only 20% of the population was registered.

## COVID-19 pandemic outbreak

As Greece was emerging from a long-lasting period of profound economic and social crisis (Tsobanoglou, 2014), with the public health care system brought to the edge of collapse, the COVID-19 outbreak took place. The first COVID-19 case was diagnosed in Greece on February 26. On March 1, the very first measure taken by the Greek government, the cancellation of the carnivals, was perceived by the public as hyperbolic, given that at the time only three cases of COVID-19 were confirmed. On March 10, with officially 89 confirmed cases and 0 deaths, all schools and universities were closed. From that very day, new regulatory measures were gradually introduced to mitigate the risk of exponential virus transmission. About 4 weeks into the pandemic, with 695 confirmed cases and 17 deaths, on March 23, nation-wide strict lockdown measures were enforced.

The general consensus for the management of this national emergency, in conjunction with management of the refugee crisis – amplified with the organized pressures by Turkey to open the border for ‘refugees’ to enter Europe – have galvanized a sense of unity and trust in the government’s directives (Dianeosis, 2020).

Thirteen hospitals have been designated as reference hospitals to deal with COVID-19 cases. Clinics have been closed and wards evacuated. Some of these have been designated for the care of infected patients, while others have been converted into COVID-19 ICU beds. Scheduled surgical operations and out-patient specialist hospital appointments have been cancelled, and only emergencies continue to be seen. The focus of the state’s attention in dealing with the COVID-19 pandemic, principally at the level of hospitals and ICU beds, as well as the current disruption of ‘regular’ services provided by the hospitals, creates the risk of a substantial increase in unmet health needs. Additionally, the partial suspension of the regular operation of the rather inadequate PHC system, along with a number of private surgeries having been forced to close because of insufficient protective equipment to continue operating, raises serious concern about access to and continuity of care. On April 4, the ‘restructuring’ of PHC services, in ways that will support a more efficiently targeted health care delivery system, was announced. Specific Health Centres in six major urban areas (Athens, Thessaloniki, Patras, Larissa, and Heraklion) have been exclusively designated for the screening of patients with respiratory infection. These COVID-19 Health Centres will be involved in early detection, monitoring, and management of possible and confirmed cases with mild symptoms that do not require hospitalization, at home, and will operate telecounseling service for these patients diagnosed with COVID-19. However, the long-standing deficiencies of the PHC system creates significant challenges for meeting the needs of vulnerable populations, for example, the homeless, drug addicts, the Roma, refugees, and other socially excluded groups, in a manner that requires coordination with welfare agencies. Currently, this is done by a plethora of civil and religious NGOs, and the local administration in a somehow haphazard manner.

One of the most sensitive and challenging issues at present relates to the appalling living conditions of approximately 40,000 asylum seekers in the refugee camps on the Aegean islands. The situation found in these camps makes it extremely difficult to take necessary precautions, such as social distancing and vigilant hygiene. Specialized medical teams were sent to the camps for the creation of self-isolation areas and compulsory temperature checking. All visits to the camps have been suspended. So far, two refugee camps on the mainland have been placed in quarantine. As of May 3, Greece with population of 10.7 million has 2620 confirmed cases, 144 deaths, 1473 recovered cases, and 37 hospitalized in ICU (https://eody.gov.gr/covid-gr-daily-report)

## COVID-19 pandemic and mental heath

The psychological impact of long-lasting strict lockdown measures and the risks linked to isolation perhaps indicate another collateral threat from the same invisible enemy of our physical health. The campaign message ‘We stay home undoubtedly has overturned everybody’s daily routine. What does this message mean for children and parents? As in any crisis, ‘renegotiation’, ‘reorganization’, and ‘reattribution of meanings’ are the new challenges that we all must face. The psychological distress caused by prolonged lockdown, along with evolving sense of inactivity, boredom, frustration, and uncertainty may lead to psychosomatic or psychological problems, alcohol drinking, dysfunctional personal and family coping strategies, increase in ineffective parent–child communication patterns. Staying at home has placed some children and adults at increased risk of domestic violence, as evidenced by increased numbers of reported incidents. However, some families have been able to mobilize resilience and functional coping strategies to manage the stress caused by home confinement and to see positive changes in their lives (e.g. spending more quality time with their children). The Greek National Public Health Organization (EODY in Greek), Public Authorities, and various civil associations have set up COVID-19 crisis hotlines.

The provision of services to people with mental health problems and their families has been substantially affected. Currently, community Adult Mental Health (AMH) and Child and Adolescent Mental Health (CAMH) services operate with reduced staff and provide for the most part telephone consultation or counseling, with only a few replacing face-to-face clinical work with Skype. Emergency cases are directed to hospitals. The operation of Day Hospital/Units has been suspended. Hospital-based AMH and CAMH services have restricted access to out-patient clinics, but continue to provide necessary treatments (e.g. depot clinic) and emergency services. In addition, psychologists provide support for colleagues on the frontline. The above measures, coupled with people’s fear of contagion, led to a substantial decline in the number of referrals or requests for assessment, even within the liaison psychiatry service. Telemental health services are advertised and provided by a large number of various professional groups, often with no supervision or use of a General Data Protection Regulation (GDPR) compliant system.

Inpatient Child and Adolescent Psychiatry (CAP) Units now apply more stringent admission criteria; only young people with severe mental health problems are admitted. In one hospital, part of the inpatient unit was closed and designated for emergency medical COVID-19 patients. This resulted in the premature discharge of patients and a shortage of CAP hospital beds. The inpatient CAP Units, and in particular their communal spaces, are not designed for physical distancing which poses a problem for patients’ care management. Daily group activity and therapeutic programs have been suspended, resulting in increased levels of distress in the young people. Policies have been changed regarding patients’ leave and parental visits, which affects the young people’s stability and, for many, feels punitive. Screening procedures for COVD-19 symptoms have been adopted but a negative test is not required before the admission. If the inpatient exhibits signs of possible infection, s/he is placed in the designated self-isolation room on the ward until the test result comes back (usually in 24–36 hours), and if the result is positive the patient is transferred to a COVID-19 designated Unit.

Parents of children with mental health problems have been advised to maintain a distant, not face-to-face contact with their clinician. It is not clear, however, what proportion of young people and their families continue to receive psychological treatments, as about 80% of services provided by the private sector (reimbursed by the National Organization for the Provision of Health Services,) have been stopped. It is safe to assume that a considerable number has ceased receiving treatment. A minority of those with developmental disorders, including autism spectrum disorders, who, prior to the COVID-19 outbreak, attended psychoeducational intervention programs, continue to receive support either through videoconferencing or through appropriate material being sent to the parents.

At this point in time, it is difficult to estimate the impact of the pandemic on the mental health of children and families. We anticipate in the long term, there is likely to be a dramatic increase in stress-related disorders, associated with increased levels of depression and anxiety in parents as a result of financial hardship. A recent survey indicated that 63% of Greeks believe that the pandemic will have a negative impact on their mental health and 57.9% on their income (Dianeosis, 2020). The increased rate of internet addiction and e-gambling among young people is another collateral threat of the pandemic.

## Learning from the pandemic

Greece as one of the countries hardest hit by the economic crisis, after 10 years of recession, dives into yet another crisis, the COVID-19 pandemic. The current situation is having a dramatic negative impact on the economy with an associated risk to people’s mental health. However, the limited resources and gaps in health system exposed during the COVID-19 outbreak give us a great opportunity to reconsider how services are organized and delivered. Perhaps now is the time to implement cardinal changes that will achieve a comprehensive and integrated health care system with procedures for horizontal and vertical connections and coordination between the different levels of care (primary, secondary, and tertiary) and the development of a long awaited electronic health record system.

It is striking that Greece with the highest per capita rate of licensed specialist physicians among EU Member States (6.2 per 1000 population) has the fourth lowest rate of health personnel employed in hospitals (Economou, 2015). The imposed freeze on hiring drove many doctors to seek work abroad or to private practice. The Greek hospital-based doctors work daily under ‘emergency’ conditions as there is no control of the patient flows given the inadequate PHC system, lack of a gate-keeping mechanism, and insufficient facilities in the provinces, that is, outside major cities. The lack of support staff, the large number of patients (the biggest hospitals in Athens receive approximately 1000 in a single 24-hour emergency shift), the over congestion on the wards (with beds placed in the corridors after the emergency shift), is a ‘normal’ work environment for the NHS hospital-based doctors, particularly during the seasonal ‘flu’, or weekends due to the endemic traffic accidents. These working conditions and large volumes, while might be seen as extraordinary for other countries, is ‘normal’ in Greece, perhaps placing the medical staff in Greece in a better position in the current pandemic crisis. Their experience of working under strenuous and very difficult conditions, with low pay and insufficient resources at their disposal, ironically might have contributed to effective management and the successful containment of cases, in conjunction with the imposed national lockdown.

The pandemic, with the introduction of ‘social distancing’, may result in dramatic changes in clinical practice, including how we deliver our treatments in the near future; and the extent to which technology can be used in safeguarding the quality of services provided.

It is important to emphasize that prior to this COVID-19 pandemic, telemedicine was available only in a few hospital-based CAMH services, with a possibility for real-time videoconferencing only with connected Health Centres or provincial hospitals. The Ministry of Health recently announced it was setting up telecounseling services for patients with COVID-19; this could become a potentially enduring tool that would allow remote monitoring at home of the elderly and those with chronic conditions and special needs. The COVID-19 pandemic is an opportunity for expanding the telemedicine system to reach out those in remote areas or islands where CAMHS are scarce and to provide consultation/guidance to PCH providers. To this end, it would be necessary in our country to enhance training in information and telecommunication technologies, alongside the development of protocols and standards, and outreach health promotion programs. In the context of tackling the pandemic, it is considered essential and urgent to develop telemedicine guidelines and to address several legal issues that will allow doctors to work without the threat of civil liability when delivering e-health care services. Telemedicine technology provides the opportunity for mental health professionals in Greece to develop effective interventions and e-therapies using digital applications, currently especially important in the circumstances imposed by the practice of social distancing. To this end, we will need to generate evidence for which types of cases telemedicine may be a preferable treatment alternative and in which instances face-to-face contact cannot be replaced.

In conclusion, the COVID-19 pandemic brought into the open the long-standing deficiencies and gaps of Greece’s under-funded public health system, due to the fundamental imbalance between public and private interests (Tountas *et al.* 2020). Weak employment security, the high number of unemployed doctors, and the sizeable informal economy situated in a ‘free’ market political system define an outdated and ultimately expensive health system. Investing in health should be seen not as a cost but as a priority social investment.

Lockdown has been a successful public health measure, but with the gradual resumption of freedom of movement and activity, every effort should be made to sustain and strengthen the health care system by enhancing community care and establishing capacity to use the ‘reserve army’ of health professionals at primary and secondary health care levels.

The pandemic, as a threat to everybody, needs to be the reason to draw back to the main Hippocratic preventive method. It will be a much-needed return to the land that developed it centuries ago (Perlstadt, 2019).
